# Prevalence of smartphone addiction and its relation with psychological distress and internet gaming disorder among medical college students

**DOI:** 10.3389/fpubh.2024.1362121

**Published:** 2024-06-03

**Authors:** Ming Zhang, Chenru Chi, Qingwei Liu, Yuhao Zhang, Xiubin Tao, Huan Liu, Bin Xuan

**Affiliations:** ^1^School of Educational Science, Anhui Normal University, Wuhu, Anhui, China; ^2^School of Innovation and Entrepreneurship, Wannan Medical College, Wuhu, Anhui, China; ^3^Graduate School of Wannan Medical College, Wuhu, Anhui, China; ^4^School of Nursing, Shandong First Medical University, Jinan, Shandong, China; ^5^School of Medical Imaging of Wannan Medical College, Wuhu, Anhui, China; ^6^Department of Nursing, The First Affiliated Hospital of Wannan Medical College (Yijishan Hospital of Wannan Medical College), Wuhu, Anhui, China; ^7^Department of Hemodialysis, The First Affiliated Hospital of Wannan Medical College (Yijishan Hospital of Wannan Medical College), Wuhu, Anhui, China

**Keywords:** smartphone addiction, psychological distress, internet gaming disorder, medical, students

## Abstract

**Background:**

The incidence of smartphone addiction has been widely studied, but the research on the relationship between smartphone addiction and psychological distress and internet gaming disorder is limited. This study investigated the characteristics and prevalence of smartphone addiction and its relation with psychological distress and internet gaming disorder. Furthermore, it provides the scientific basis for intervention measures in schools, families, and society.

**Methods:**

A random cluster sampling method was applied to investigate 656 medical students from grades 1 to 4 at Wannan Medical College in Anhui province, People’s Republic of China. The questionnaire consisted of general information, a smartphone addiction scale, an Internet gaming disorder scale, and a Kessler 6-item psychological distress test. The obtained results were first summarized using descriptive statistics. The Chi-square test was used to compare the status of smartphone addiction. Binary logistic regression was used to analyze the relationship between smartphone addiction and various variables.

**Results:**

Our results showed that the prevalence of smartphone addiction in medical students was 49.5% (325/656). Psychological distress (*p* < 0.001), internet gaming disorder (*p* < 0.001), and childhood trauma (*p* = 0.001) were highly correlated with smartphone addiction in medical students. Psychological distress, and internet gaming disorder were positively associated with smartphone addiction (*p* < 0.000).

**Conclusion:**

The prevalence of smartphone addiction is high among medical students in Chinese. Smartphone addiction is highly related to related to internet gaming disorder and psychological distress.

## Introduction

The smartphone represents the most significant technological advance of the 21st century (1). The increasingly rich functions of smartphones have brought great convenience to our life, study and work, and at the same time, it has also caused many increasingly serious social problems. According to the Cyberspace Administration of China (2), by December 2022, the number of Internet users in China reached 1.067 billion, an increase of 35.49 million over December 2021, of which 99.8% of Chinese Internet users use mobile phones to access the Internet. However, just like every coin has two sides, smart phones also have some obvious disadvantages, smart phones are a double-edged sword. Studies have shown the associations between smartphone addiction and individual health outcomes, such as mental health (3, 4), sleep disturbances (5, 6) and quality of life (7). Research has shown that smartphone addiction can lead to a variety of problems, such as anxiety, depression, sleep disorders, mood disorders, social disorders, and even suicide (8–10). Today’s college students are growing up with smartphones, which have become a necessity for college student’s life and study (11). Compared with other professions in society, college students have more access to the Internet, prefer to establish online relationships, and are more likely to develop symptoms of smartphone addiction (12). One meta-analysis found that the average prevalence of smartphone addiction among Chinese college students was about 23% (13). During the COVID-19 pandemic, smartphones have played a considerable role in medical care and higher education due to their powerful functions (14), making college students more dependent on the Internet and smartphones in life and study (14). A national survey of 746,217 Chinese college students by Ma et al. (15) found that the prevalence of acute stress, anxiety and depressive symptoms among college students was 34.9, 11, and 21.1%, and the risk of depression and anxiety disorders increased with the increase of electronic device exposure time. One study found that problematic smartphone use was associated with fatigue symptoms and problems with sleep quality in medical students (16).

Psychological distress refers to symptoms such as anxiety, depression, psychological stress, and absence of well-being (17). Higher levels of problematic Internet use among student nurses have been confirmed to be associated with increased psychological distress (18). Psychological distress is highly correlated with burnout, cognitive problems, and behavioral problems (19). A study (20) found a high rate of self-reported psychological distress among school-aged children and adolescents during the COVID-19 pandemic and a significant correlation between Internet-related behaviors and psychological distress among Chinese children during the COVID-19 pandemic.

Internet Gaming Disorder (IGD) is an activity characterized by persistent and repeated Internet use to play video games over 1 year, which can lead to significant considerable impairment or distress to the individual (21). Symptoms in people with IGD include excessive addiction to video games, significant withdrawal symptoms, unsuccessful attempts to stop, and other symptoms similar to substance dependence jeopardizing meaningful relationships or opportunities due to video games (22). IGD has become a substantial and widespread public health threat worldwide. In addition to the symptoms of smartphone addiction, it is also important to carefully assess the risk of smartphone addiction, which is essential for future prevention and intervention measures. Study showed that IGD is an emerging health issue for men (23). Studies also found that men risk developing IGD more than women (24–26). Teng et al. (27) found that IGD is negatively correlated with self-esteem and social support. Given these current limitations, we believe further research is warranted to explore the relationship between psychological distress, IGD, and smartphone addiction.

Many mechanistic studies have explored the relationship between smartphone addiction and mental health. Smartphone addiction can cause symptoms of depression, anxiety, and loneliness, and affect individual’s mental health through a variety of complex mechanisms (28). There are several theories to explain the development of smartphone addiction (29–31). For example, the Person-Affect-Cognition-Execution (I-PACE) model suggests that individuals could use smartphones as a coping strategy to overcome their troubles and satisfy their emotional needs. However, when individuals use their smartphones too often to form habitual and dependent behaviors, they risk developing smartphone addiction symptoms. However, the mechanism of the development of smartphone addiction is still unclear, because the factors affecting behavioral addiction are very complex, and more research evidence is needed to confirm these theories and models. In this sense, it is more urgent and important to explore the relationship between smartphone addiction, psychological distress, and online gaming.

Childhood trauma is defined as experiences of extreme threats experienced or perceived by children early in life, including the death of a parent, exposure to war, harrowing accidents, serious illness, exposure to violence, childhood neglect, and abuse (32, 33). The incidence of childhood trauma is high, with approximately two-thirds of the population experiencing severe childhood trauma before 18 years old (34). Research (35) had found that childhood trauma often leads to many harmful health outcomes. Studies have found that childhood trauma is extremely harmful and can lead to various psychological and behavioral problems in individuals, including depression, increased aggression, substance abuse, interpersonal difficulties, PTSD, and even suicidal thoughts (36, 37). Research has found that compared to college students who have not been abused, college students who were abused in childhood are more likely to induce mobile phone addiction (38). One study confirmed that experiencing trauma had a significant predictive effect on smartphone addiction among college students (39). Studies have found that childhood trauma greatly increases the risk of PSH in adolescents, and childhood trauma is also highly associated with multiple types of mental disorders, affecting them into adulthood (40).

Therefore, smartphone addiction and mental health problems among medical students should attract more attention. Based on the existing theoretical mechanisms and research, smartphone addiction seems to be related to IGD and psychological distress among medical students. We thus proposed the following research hypotheses:

*Hypothesis 1 (H1)*: Smartphone addiction is positively correlated with levels of IGD and psychological distress.

*Hypothesis 2 (H2)*: Childhood trauma significantly and positively predicts smartphone addiction among medical college students.

## Materials and methods

### Study design and participants

This study conducted a cross-sectional survey at Wannan Medical College in Wuhu City, southern Anhui Province, China, from June to July 2023. The method of cluster sampling was used to investigate Wannan Medical College in Wuhu City. The research team conducted detailed training for the investigators in advance to ensure the quality of the investigation after selecting counselors and class cadres as investigators. Before the survey, students were informed about the purpose and measurement method of the survey, and the paper questionnaire was issued after obtaining their signed consent. It took about 5–10 min to complete all the questions and can be recalled after the investigators have checked it. The investigator from each college was trained in standardised questionnaires collection, sending and receiving paper versions of the questionnaire, and all participants volunteered to participate in the study and signed paper informed consent forms.

A cluster sampling survey was used to recruit participants. Four different grades were randomly selected, and the inclusion criteria for participants were: (1) students in the Wannan Medical College, (2) agreed to participate and signed a paper informed consent form. The exclusion criteria were: (1) dropped out of Wannan Medical College; (2) did not complete the questionnaire; (3) whose response time is more than 20 min.

**Figure 1:**
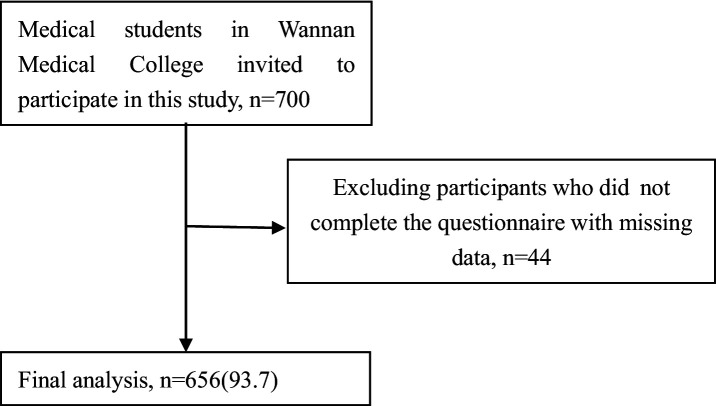
Sample selection process for this cross-sectional study.

A total of 700 medical students completed the investigation, of which 44 were excluded, and the remaining 656 respondents met the requirements, with an effective response proportion of 93.7% (details are shown in Figure 1).

### Measurement

#### General demographic characteristics

Personal information of participants was collected using a self-reported questionnaire. The contents of the questionnaire include age, gender, grade, place of origin (rural/urban/urban), monthly living expenses, academic pressure, the only child, serving as a student leader (yes/no), playing online games (yes/no), whether you feel lonely, etc.

#### Smartphone addiction scale short version (SAS-SV)

The Smartphone Addiction Scale Short version (SAS-SV) (41) was used to measure the smartphone addiction status of medical students. SAS-SV consists of 10 items with a scale of Likert 6 scales (1 = strongly disagree, 6 = strongly agree). The total score ranges from 10 to 60. The smartphone addiction thresholds for male and female subjects were ≥ 31 and ≥ 33, respectively. The Cronbach’s alpha coefficient in this study was 0.81. The SAS-SV has been confirmed to have good reliability and validity among Chinese medical students (42). In this study, the Cronbach’s α coefficient of SAS-SV was 0.83.

#### Kessler 6-item psychological distress scale (K-6)

Psychological distress was measured via the Kessler 6-item psychological distress scale (K-6). The K-6 scale was developed by Kessler et al. (43). It has been proven and widely used to assess an individual’s degree of non-specific psychological distress within the past 1 month. The Chinese version was proved to have good reliability and validity (44). The scale consists of six psychological symptoms. The scale is scored by a 5-point Likert scale (0 = no time, 4 = all the time). The total score is 0 ~ 24, and more than 12 points were classified as severe psychological distress (45). In this study, the Cronbach’s α coefficient of K-6 was 0.79.

#### IGD

To evaluate the symptoms of IGD among medical students, the diagnostic criteria in the Diagnostic and Statistical Manual of Mental Disorders Fifth Edition (DSM-5) (45) were used. The scale comprises nine items, and participants were asked whether these symptoms (e.g., enthusiasm, withdrawal, tolerance, loss of control, loss of interest in other activities, persistence, cheating, avoidance, victimization) occurred in the past 12 months (0 = no, 1 = yes). A total of five positive responses was suggestive of a higher risk of IGD (46). In this study, the Cronbach’s coefficient of the scale was 0.72.

### Statistical analysis

Data analyses were performed using IBM Statistical Package for Social Science, Version 21.0 (SPSS Inc., Chicago, IL, United States). Descriptive statistical methods were used to summarize participants’ characteristics. Based on the Smartphone Addiction Scale score, the participants were divided into two groups: non-smartphone addiction and smartphone addiction. Chi-square tests were used to examine differences in demographics, psychological distress, and IGD symptoms between the smartphone and non-smartphone addiction groups. Binary logistic regression analysis was used to analyze the factors associated with smartphone addiction, and the ORs (odds ratios) and 95% CIs (confidence intervals) were calculated.

## Result

### Participant characteristics

Among 656 medical student included in the data analysis, The age of the respondents ranged from 17 to 24 years old, which the mean age being (19.66 ± 2.14) years old. 388 (59.1%) were male, and 268 (40.9%) were female. 390 (59.5%) were the only child, 291(44.3%) were freshmen, 49(7.5%) were sophomore, 257(39.2%) were junior, and 59(9.0) were senior. Further socio-demographic information about this study is displayed in Table 1.

**Table 1:** Participants’ demographic information (*n* = 656).

Item	Category	Participants	Percentage (%)
Gender	Male	388	59.1
	Female	268	40.9
The only child	No	390	59.5
	Yes	266	40.5
Grade	Freshmen	291	44.3
	Sophomore	49	7.5
	Junior	257	39.2
	Senior	59	9.0
Student leader	No	392	59.8
	Yes	264	40.2
Place of residence	Rural	267	40.7
	Town	230	35.1
	City	159	24.2
Want to get scholarship	No	254	38.7
	Yes	402	61.3
Smoking	No	537	81.8
	Already quit smoking	51	7.8
	Yes	68	10.4
Want to change major	No	522	79.6
	Yes	134	20.4
Drink alcoholic	No	397	60.5
	Already quit drinking	66	10.1
	Yes	193	29.4
Satisfied with the major	No	101	15.4
	Generally	272	41.5
	Yes	283	43.1
Childhood trauma	No	468	71.3
	Yes	188	28.7
College adaptability	Not adapt	110	16.8
	Generally	243	37.0
	Adapt	303	46.2
Study stress	Low	149	22.7
	Generally	311	47.4
	High	196	29.9
Loneliness	No	301	45.9
	Generally	260	39.6
	Yes	95	14.5
Monthly living expenses	≤1,499	149	22.7
	1,500 ~ 1999	281	42.8
	2000 ~ 2,499	189	28.8
	≥2,500	37	5.7
IGD	No	527	80.3
	Yes	129	19.7
Psychological distress	No	554	84.5
	Yes	102	15.5

### Factors associated with smartphone addiction in the univariate analysis

In this study, the prevalence of smartphone addiction among the medical students was 49.5% (325/656). There were significant differences between the the only child or not, grade, want to change major, satisfied with the major, childhood trauma, college adaptability, study stress, monthly living expenses, IGD, and psychological distress (K-6) (*p* < 0.05, Table 2).

**Table 2:** Characteristics of the participants based on the presence of smartphone addiction (*n* = 656).

	Non-Smartphone addiction (*n* = 331)	Smartphone addiction (*n* = 325)	χ^2^	*p*
Gender			0.125	0.724
Male	198(51.0)	190(49.0)		
Female	133(49.6)	135(50.4)		
The only child			5.113	0.024
No	211(54.1)	179(45.9)		
Yes	120(45.1)	146(54.9)		
Grade			14.484	0.002
Freshmen	144(49.5)	147(50.5)		
Sophomore	19(38.8)	30(61.2)		
Junior	148(57.6)	109(42.4)		
Senior	20(33.9)	39(66.1)		
Student leader			1.708	0.191
No	206(52.6)	186(47.4)		
Yes	125(47.3)	139(52.7)		
Place of residence			2.567	0.277
Rural	136(50.9)	131(49.1)		
Town	123(53.5)	107(46.5)		
City	72(45.3)	87(54.7)		
Want to change major			0.379	0.538
No	132(52.0)	122(48.0)		
Yes	199(49.5)	203(50.5)		
Want to change major			5.968	0.015
No	276(52.9)	246(47.1)		
Yes	55(41.0)	79(59.0)		
Smoking			4.559	0.102
No	278(51.8)	259(48.2)		
Already quit smoking	27(52.9)	24(47.1)		
Yes	26(38.2)	42(61.8)		
Drink alcoholic			5.672	0.059
No	215(54.2)	182(45.8)		
Already quit drinking	31(47.0)	35(53.0)		
Yes	85(44.0)	108(56.0)		
Satisfied with the major			8.078	0.018
No	48(47.5)	53(52.5)		
Generally	155(57.0)	117(43.0)		
Yes	128(45.2)	155(54.8)		
Childhood trauma			30.275	<0.001
No	268(57.3)	200(42.7)		
Yes	63(33.5)	125(66.)		
College adaptability			5.870	0.053
No	44(40.0)	66(60.0)		
Generally	126(51.9)	117(48.1)		
Yes	161(53.1)	142(46.9)		
Study stress			25.523	<0.001
No	79(53.0)	70(47.0)		
Generally	182(58.5)	129(41.5)		
Yes	70(35.7)	126(64.3)		
Loneliness			3.939	0.140
No	163(54.2)	138(45.8)		
Generally	127(48.8)	133(51.2)		
Yes	41(43.2)	54(56.8)		
Monthly living expenses			19.938	<0.001
≤1,499	92(61.7)	57(38.3)		
1,500 ~ 1999	149(53.0)	132(47.0)		
2,000 ~ 2,499	72(38.1)	117(61.9)		
≥2,500	18(48.6)	19(51.4)		
IGD			108.799	<0.001
No	319(60.5)	208(39.5)		
Yes	12(9.3)	117(90.7)		
Psychological distress			43.106	<0.001
No	310(56.0)	244(44.0)		
Yes	21(20.6)	81(79.4)		

### Correlation between smartphone addiction and psychological distress and IGD in medical college students

**Figure 2:**
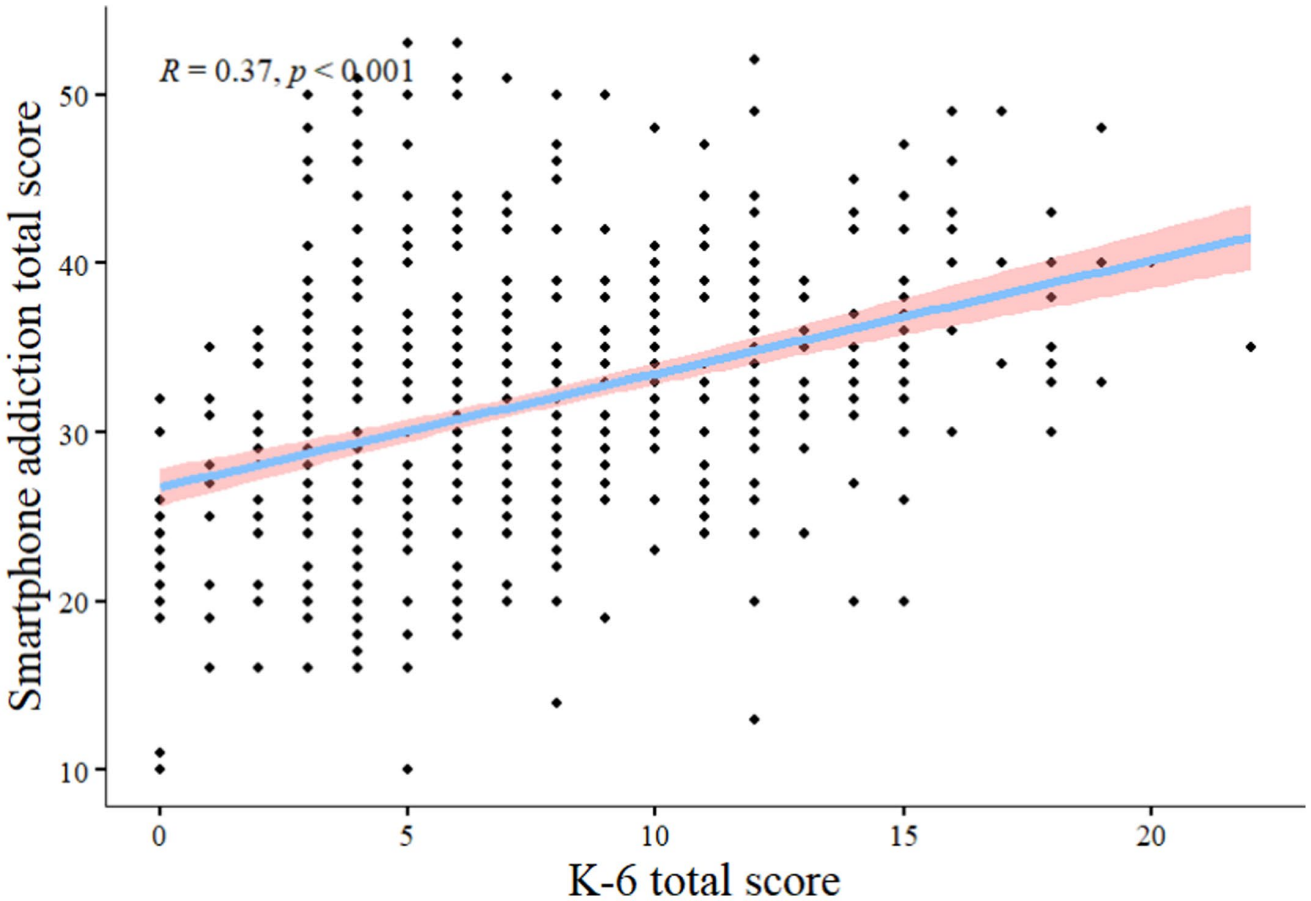
Smartphone addiction total score vs. K-6 total score Pearson Correlation.

**Figure 3:**
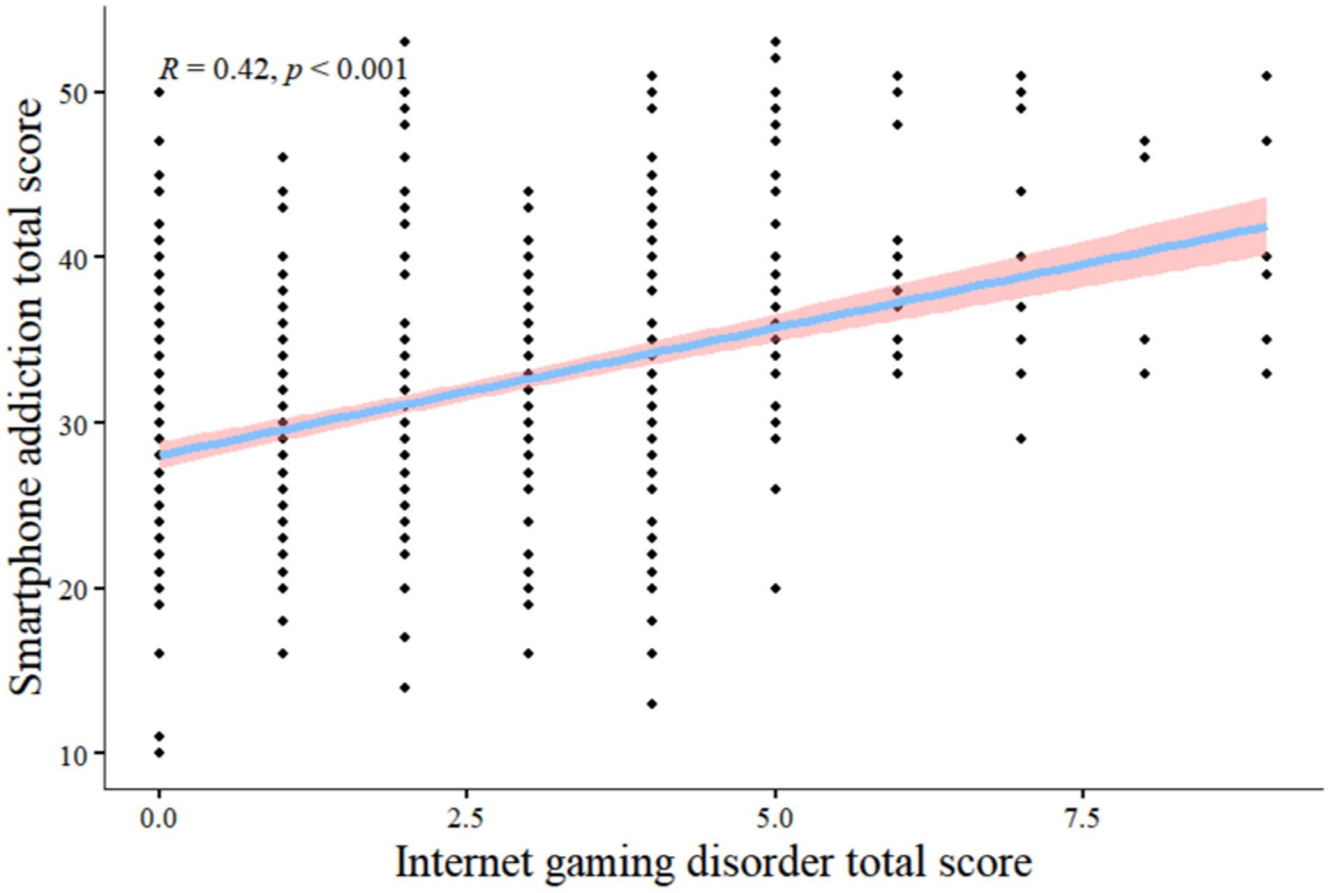
Smartphone addiction total score vs. internet gaming disorder total score Pearson Correlation.

From Figures 2, 3 psychological distress, and IGD were positively associated with smartphone addiction (*r* = 0.37, *P*<0.001; *r* = 0.42, *P*<0.001).

### Binary analysis factors associated with smartphone addiction

**Figure 4:**
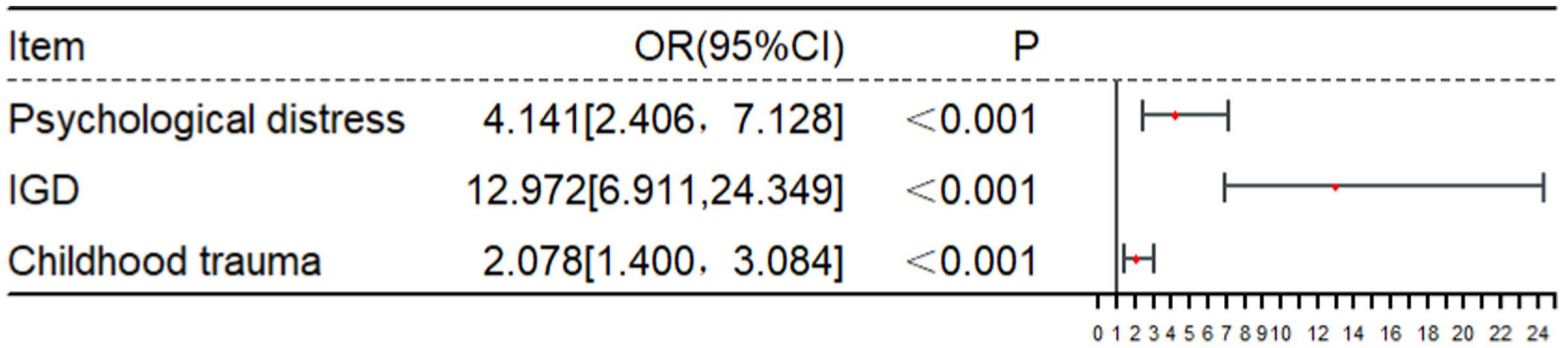
Forest plot: factors affecting smartphone addiction using binary logistic regression analysis.

We put independent variables (*p* < 0.05) and dependent variables (0 = non-smartphone addiction, 1 = smartphone addiction) into a binary logistic regression analysis model. Factors affecting smartphone addiction of medical students are shown in Table 3. As shown in Table 3 and Figure 4, smartphone addiction is more severe among medical students with psychological distress, IGD, and childhood trauma(OR = 4.275, 95% CI 2.475–7.383; OR = 13.010, 95% CI 6.923–24.449; OR = 2.000, 95% CI 1.344–2.976).

**Table 3:** Binary logistic regression analysis of factors associated with smartphone addiction.

Variable	B	SE	Wald	*p*	OR	95% CI
Psychological distress	1.421	0.277	26.298	<0.001	4.141	2.406–7.128
IGD	2.563	0.321	63.626	<0.001	12.972	6.911–24.349
Childhood trauma	0.731	0.202	13.173	<0.001	2.078	1.400–3.084
Constant	−0.803	0.111	52.044	<0.001	0.448	

## Discussion

### Key findings

With the progress of science and technology, smart phones have become an increasingly important and indispensable part of college students’ lives. In recent years, the overuse of smart phones has become a public health problem of widespread concern (47). The purpose of this study was to explore the status of smartphone addiction among medical students and its relationship with IGD and psychological distress. The results showed that the smartphone addiction rate among medical students is relatively high, which is basically consistent with previous research results (11). According to the SAS-SV scale, the smartphone addiction rate among students in different countries ranged from 12 to 78.3% (48). This difference may stem from the differences in different social and cultural environments and the development of information technology. At the same time, the measurement scales used in different studies may differ.

### Differences in smartphone addiction

Consistent with previous studies (49–51), this study found that Psychological distress is one of the important risks leading to smartphone addiction among medical students. Studies have found significant associations between smartphone addiction and psychosocial factors such as depression, anxiety, and stress among university medical students (48, 52). Study (53) found that psychological distress indirectly affects smartphone addiction by affecting social capital and social need satisfaction. Psychological distress is a negative emotion that is an indicator of poor levels of mental health, with symptoms including anxiety, depression, behavioral problems, and functional impairment (54). Studies have found strong correlations between psychological distress and smartphone addiction among university students (55–57). The above studies supported our Hypothesis 1 (H1), that smartphone addiction is positively correlated with psychological distress.

This study found that IGD is one of the important influencing factors of smartphone addiction. Research shows that the prevalence of IGD has increased in recent years (58). Research has proven that IGD has been associated with loneliness and introversion, boredom tendencies, social inhibition, and decreased self-control ability (59). Internet gaming is a popular leisure activities among college students, while dysfunctional gaming can lead to addiction-like symptoms. A systematic review of relevant studies in China showed that the prevalence of gaming disorder ranges from 3.5 to 17% (60). Another study found that the prevalence of IGD in men is higher than that in women (61). Studies shown that heavy gamers had lower levels of self-control (62) and, in addition, higher levels of impulsivity (63, 64). Research shown that excessive addiction to online games is a common feature among some college students and is also a decisive factor in their Internet addiction (65). The above studies supported our Hypothesis 1 (H1), that smartphone addiction is positively correlated with levels of IGD.

Exposure to childhood trauma is a risk factor for psychosis (66). Research has found that childhood trauma is linked to poorer physical and mental health outcomes in adulthood (67, 68). Childhood trauma could increase the risk of physical and mental health problems in adults and unhappiness (69, 70). Adolescents who grew up in unsafe family environments and suffered childhood trauma may self-medicate negative emotions through excessive use of the Internet, ultimately leading to Internet addiction (IA) (71). For individuals who have experienced childhood trauma, excessive use of the Internet is a strategy to cope with stress. However, this strategy further strengthens their dependence on the Internet and is prone to Internet addiction (71). The above studies supported our Hypothesis 2 (H2), that childhood trauma is one of the influencing factors of smartphone addiction.

### Limitations

This study still has some limitations. First, the study used a cross-sectional design, so causal relationships between the study variables could not be determined. Future prospective studies should be conducted on serial assessments of smartphone addiction causes and psychological stress and online gaming addiction. Second, our study sample only included medical students from one medical school in Anhui Province, so generalization of the results to the whole of China or other countries would be limited. Third, the sample size is limited. Finally, because the questionnaire method is based on the subjective evaluation of the research subjects, the participants’ answers may be exaggerated or weakened.

## Conclusion

In summary, smartphone addiction is related to psychological distress and IGD. For medical students with psychological distress, IGD, and childhood trauma, educational institutions and teachers should recognize early and provide relevant psychological assistance and intervention measures to prevent their further development, thereby reducing smartphone addiction. Additionally, teachers should consider childhood trauma and psychological distress when dealing with smartphone-addicted medical students, which could help provide more effective interventions.

## Data availability statement

The raw data supporting the conclusions of this article will be made available by the authors, without undue reservation.

## Ethics statement

All students were informed of the purpose of the study and signed written consent. Ethical approval was obtained from the Ethics Committee of Wannan Medical College (Decision No. 2023212).

## Author contributions

MZ: Funding acquisition, Investigation, Resources, Supervision, Writing – original draft, Writing – review & editing. CC: Formal analysis, Investigation, Writing – original draft. QL: Data curation, Investigation, Software, Writing – original draft. YZ: Investigation, Methodology, Validation, Writing – review & editing. XT: Formal analysis, Investigation, Resources, Writing – review & editing. HL: Data curation, Formal analysis, Investigation, Software, Validation, Writing – original draft, Writing – review & editing. BX: Conceptualization, Methodology, Supervision, Validation, Writing – original draft, Writing – review & editing.

## References

[1] Moses JC Adibi S Wickramasinghe N Nguyen L Angelova M Islam SMS. Smartphone as a disease screening tool: a systematic review. Sensors. (2022) 22:3787. doi: 10.3390/s22103787, PMID: 35632195 PMC9145643

[2] China CAO (2023) China CAo: The 51th China statistical report on internet development. Available at: https://cnnic.cn/n4/2023/0302/c199-10755.html

[3] Chen IH Chen CY Liu CH Ahorsu DK Griffiths MD Chen YP. Internet addiction and psychological distress among Chinese schoolchildren before and during the COVID-19 outbreak: a latent class analysis. J Behav Addict. (2021) 10:731–46. doi: 10.1556/2006.2021.00052, PMID: 34529588 PMC8997210

[4] Kakul F Javed S. Internet gaming disorder: an interplay of cognitive psychopathology. Asian J Soc Health Behav. (2023) 6:36–45. doi: 10.4103/shb.shb_209_22

[5] Chang KC Chang YH Yen CF Chen JS Chen PJ Lin CY. A longitudinal study of the effects of problematic smartphone use on social functioning among people with schizophrenia: mediating roles for sleep quality and self-stigma. J Behav Addict. (2022) 11:567–76. doi: 10.1556/2006.2022.00012, PMID: 35394922 PMC9295235

[6] Ranjan LK Gupta PR Srivastava M Gujar NM. Problematic internet use and its association with anxiety among undergraduate students. Asian J Soc Health Behav. (2021) 4:137–41. doi: 10.4103/shb.shb_30_21

[7] Kwok C Leung PY Poon KY Fung XC. The effects of internet gaming and social media use on physical activity, sleep, quality of life, and academic performance among university students in Hong Kong: a preliminary study. Asian J Soc Health Behav. (2021) 4:36–44. doi: 10.4103/shb.shb_81_20

[8] Cho HY Kim DJ Park JW. Stress and adult smartphone addiction: mediation by self-control, neuroticism, and extraversion. Stress Health. (2017) 33:624–30. doi: 10.1002/smi.2749, PMID: 28332778

[9] Yang H Liu B Fang J. Stress and problematic smartphone use severity: smartphone use frequency and fear of missing out as mediators. Front Psych. (2021) 12:659288. doi: 10.3389/fpsyt.2021.659288, PMID: 34140901 PMC8203830

[10] Kil N Kim J McDaniel JT Kim J Kensinger K. Examining associations between smartphone use, smartphone addiction, and mental health outcomes: a cross-sectional study of college students. Health Promot Perspect. (2021) 11:36–44. doi: 10.34172/hpp.2021.06, PMID: 33758754 PMC7967133

[11] Liu H Zhou Z Huang L Zhu E Yu L Zhang M. Prevalence of smartphone addiction and its effects on subhealth and insomnia: a cross-sectional study among medical students. BMC Psychiatry. (2022) 22:305. doi: 10.1186/s12888-022-03956-6, PMID: 35488216 PMC9052183

[12] Jang KS Hwang SY Choi JY. Internet addiction and psychiatric symptoms among Korean adolescents. J Sch Health. (2008) 78:165–71. doi: 10.1111/j.1746-1561.2007.00279.x 18307612

[13] Tao J Luo C Huang J. Meta-analysis of the current situation of mobile phone dependence among college students in China (in Chinese). Chin J School Health. (2018) 39:1391–4. doi: 10.16835/j.cnki.1000-9817.2018.09.032

[14] Iyengar K Upadhyaya GK Vaishya R Jain V. COVID-19 and applications of smartphone technology in the current pandemic. Diabetes Metab Syndr. (2020) 14:733–7. doi: 10.1016/j.dsx.2020.05.033, PMID: 32497963 PMC7248636

[15] Ma Z Zhao J Li Y Chen D Wang T Zhang Z. Mental health problems and correlates among 746 217 college students during the coronavirus disease 2019 outbreak in China. Epidemiol Psychiatr Sci. (2020) 29:e181. doi: 10.1017/S2045796020000931, PMID: 33185174 PMC7681173

[16] Zhang C Zeng P Tan J Sun S Zhao M Cui J. Relationship of problematic smartphone use, sleep quality, and daytime fatigue among quarantined medical students during the COVID-19 pandemic. Front Psych. (2021) 12:755059. doi: 10.3389/fpsyt.2021.755059, PMID: 34858229 PMC8631394

[17] Burnette JL Knouse LE Vavra DT O'Boyle E Brooks MA. Growth mindsets and psychological distress: a meta-analysis. Clin Psychol Rev. (2020) 77:101816. doi: 10.1016/j.cpr.2020.101816, PMID: 32163802

[18] Labrague LJ. Problematic internet use and psychological distress among student nurses: the mediating role of coping skills. Arch Psychiatr Nurs. (2023) 46:76–82. doi: 10.1016/j.apnu.2023.08.009, PMID: 37813508

[19] Arbabisarjou A Mehdi HS Sharif MR Alizadeh KH Yarmohammadzadeh P Feyzollahi Z. The relationship between sleep quality and social intimacy, and academic burn-out in students of medical sciences. Glob J Health Sci. (2015) 8:231–8. doi: 10.5539/gjhs.v8n5p231, PMID: 26652080 PMC4877193

[20] Chen IH Chen C-Y Pakpour AH Griffiths MD Lin CY. Internet-related behaviors and psychological distress among schoolchildren during COVID-19 school suspension. J Am Acad Child Adolesc Psychiatry. (2020) 59:1099–1102.e1. doi: 10.1016/j.jaac.2020.06.007, PMID: 32615153 PMC7833594

[21] King DL Delfabbro PH. The cognitive psychology of internet gaming disorder. Clin Psychol Rev. (2014) 34:298–308. doi: 10.1016/j.cpr.2014.03.006 24786896

[22] Wu AMS Chen JH Tong KK Yu S Lau JTF. Prevalence and associated factors of internet gaming disorder among community dwelling adults in Macao China. J Behav Addict. (2018) 7:62–9. doi: 10.1556/2006.7.2018.12, PMID: 29463097 PMC6035015

[23] Chen KH Oliffe JL Kelly MT. Internet gaming disorder: an emergent health issue for men. Am J Mens Health. (2018) 12:1151–9. doi: 10.1177/1557988318766950, PMID: 29606034 PMC6131461

[24] Yu Y Mo PKH Zhang J Li J Lau JTF. Why is internet gaming disorder more prevalent among Chinese male than female adolescents? The role of cognitive mediators. Addict Behav. (2021) 112:106637. doi: 10.1016/j.addbeh.2020.106637, PMID: 32919322

[25] Wu XS Zhang ZH Zhao F Wang WJ Li YF Bi L. Prevalence of internet addiction and its association with social support and other related factors among adolescents in China. J Adolesc. (2016) 52:103–11. doi: 10.1016/j.adolescence.2016.07.012 27544491

[26] Taechoyotin P Tongrod P Thaweerungruangkul T Towattananon N Teekapakvisit P Aksornpusitpong C. Prevalence and associated factors of internet gaming disorder among secondary school students in rural community, Thailand: a cross-sectional study. BMC Res Notes. (2020) 13:11. doi: 10.1186/s13104-019-4862-3, PMID: 31907063 PMC6945594

[27] Teng ZJ Pontes HM Nie Q Guo C. Internet gaming disorder and psychosocial well-being: a longitudinal study of older-aged adolescents and emerging adults. Addict Behav. (2020) 110:106530. doi: 10.1016/j.addbeh.2020.106530, PMID: 32683173

[28] Park N Lee H. Social implications of smartphone use: Korean college students' smartphone use and psychological well-being. Cyberpsychol Behav Soc Netw. (2012) 15:491–7. doi: 10.1089/cyber.2011.0580, PMID: 22817650

[29] Brand M Wegmann E Stark R Müller A Wölfling K Robbins TW. The interaction of person-affect-cognition-execution (I-PACE) model for addictive behaviors: update, generalization to addictive behaviors beyond internet-use disorders, and specification of the process character of addictive behaviors. Neurosci Biobehav Rev. (2019) 104:1–10. doi: 10.1016/j.neubiorev.2019.06.032, PMID: 31247240

[30] Campbell JL Miller JD. Narcissism and the world wide web In: Campbell WK Miller JD, editors. The handbook of narcissism and narcissistic personality disorder: Theoretical approaches, empirical findings, and treatments. Hoboken, NJ: John Wiley & Sons, Inc (2011). 371–81.

[31] McCain JL Campbell WK. Narcissism and social media use: a meta-analytic review. Psychol Pop Media Cult. (2018) 7:308–27. doi: 10.1037/ppm0000137

[32] American Psychiatric Association. Diagnostic and statistical manual of mental disorders. 4th ed. Washington, DC: American Psychiatric Association (2001).

[33] Bulut S. Classification of posttraumatic stress disorder and its evolution in diagnostic and statistical manual of mental disorders (DSM) criteria. Int J Psych Counsell. (2020) 12:105–8. doi: 10.5897/IJPC2020.0597

[34] Hughes K Bellis MA Hardcastle KA Sethi D Butchart A Mikton C. The effect of multiple adverse childhood experiences on health: a systematic review and meta-analysis. Lancet Public Health. (2017) 2:e356–66. doi: 10.1016/S2468-2667(17)30118-4, PMID: 29253477

[35] Gladish N Merrill SM Kobor MS. Childhood trauma and epigenetics: state of the science and future. Curr Environ Health Rep. (2022) 9:661–72. doi: 10.1007/s40572-022-00381-5, PMID: 36242743

[36] El-Khodary B Samara M. The relationship between multiple exposures to violence and war trauma, and mental health and behavioural problems among Palestinian children and adolescents. Eur Child Adolesc Psychiatry. (2020) 29:719–31. doi: 10.1007/s00787-019-01376-8, PMID: 31352503

[37] Nelson CA Scott RD Bhutta ZA Harris NB Danese A Samara M. Adversity in childhood is linked to mental and physical health throughout life. BMJ. (2020) 371:m3048. doi: 10.1136/bmj.m3048, PMID: 33115717 PMC7592151

[38] Schwandt ML Heilig M Hommer DW George DT Ramchandani VA. Childhood trauma exposure and alcohol dependence severity in adulthood: mediation by emotional abuse severity and neuroticism. Alcohol Clin Exp Res. (2013) 37:984–92. doi: 10.1111/acer.12053, PMID: 23278300 PMC3620963

[39] Liang HY Zhang B Jiang HB Zhou HL. Adult attachment: its mediation role on childhood trauma and mobile phone addiction. J Psychol Africa. (2021) 31:369–74. doi: 10.1080/14330237.2021.1952706

[40] Green JG McLaughlin KA Berglund PA Gruber MJ Sampson NA Zaslavsky AM. Childhood adversities and adult psychiatric disorders in the national comorbidity survey replication I: associations with first onset of DSM-IV disorders. Arch Gen Psychiatry. (2010) 67:113–23. doi: 10.1001/archgenpsychiatry.2009.186, PMID: 20124111 PMC2822662

[41] Kwon M Kim DJ Cho H Yang S. The smartphone addiction scale: development and validation of a short version for adolescents. PLoS One. (2013) 8:e83558. doi: 10.1371/journal.pone.0083558, PMID: 24391787 PMC3877074

[42] Liu H Zhou Z Zhu E Huang L Zhang M. Smartphone addiction and its associated factors among freshmen medical students in China: a cross-sectional study. BMC Psychiatry. (2022) 22:308. doi: 10.1186/s12888-022-03957-5, PMID: 35501728 PMC9058751

[43] Kessler RC Andrews G Colpe LJ Hiripi E Mroczek DK Normand SLT. Short screening scales to monitor population prevalences and trends in non-specific psychological distress. Psychol Med. (2002) 32:959–76. doi: 10.1017/S0033291702006074, PMID: 12214795

[44] Kang YK Guo WJ Xu H Chen YH Li XJ Tan ZP. The 6-item Kessler psychological distress scale to survey serious mental illness among Chinese undergraduates: psychometric properties and prevalence estimate. Compr Psychiatry. (2015) 63:105–12. doi: 10.1016/j.comppsych.2015.08.011, PMID: 26555498

[45] American Psychiatric Association. Diagnostic and statistical manual of mental disorders. DSM-5. 5th edn. Washington, DC: American Psychiatric Publishing (2013). P. 371.

[46] Petry NM Rehbein F Gentile DA Lemmens JS Rumpf HJ Mößle T. An international consensus for assessing internet gaming disorder using the new DSM-5 approach. Addiction. (2014) 109:1399–406. doi: 10.1111/add.12457, PMID: 24456155

[47] Shoukat S. Cell phone addiction and psychological and physiological health in adolescents. Excli J. (2019) 18:47–50. PMID: 30956638 PMC6449671

[48] Nikolic A Bukurov B Kocic I Vukovic M Ladjevic N Vrhovac M. Smartphone addiction, sleep quality, depression, anxiety, and stress among medical students. Front Public Health. (2023) 11:1252371. doi: 10.3389/fpubh.2023.1252371, PMID: 37744504 PMC10512032

[49] Lei LY Ismail MA Mohammad JA Yusoff MSB. The relationship of smartphone addiction with psychological distress and neuroticism among university medical students. BMC Psychol. (2020) 8:97. doi: 10.1186/s40359-020-00466-6, PMID: 32917268 PMC7488412

[50] Ou-Yang Q Liu Q Song PY Wang JW Yang S. The association between academic achievement, psychological distress, and smartphone addiction: a cross-sectional study among medical students. Psychol Health Med. (2023) 28:1201–14. doi: 10.1080/13548506.2022.2148697 36411542

[51] Alzhrani AM Aboalshamat KT Badawoud AM Abdouh IM Badri HM Quronfulah BS. The association between smartphone use and sleep quality, psychological distress, and loneliness among health care students and workers in Saudi Arabia. PLoS One. (2023) 18:e0280681. doi: 10.1371/journal.pone.0280681, PMID: 36701337 PMC9879389

[52] Zhang K Guo H Wang T Zhang J Yuan G Ren J. A bidirectional association between smartphone addiction and depression among college students: a cross-lagged panel model. Front Public Health. (2023) 11:1083856. doi: 10.3389/fpubh.2023.1083856, PMID: 36761134 PMC9902510

[53] Bian M Leung L. Linking loneliness, shyness, smartphone addiction symptoms, and patterns of smartphone use to social capital. Soc Sci Comput Rev. (2015) 33:61–79. doi: 10.1177/0894439314528779

[54] Drapeau A Marchand A Beaulieu-Prévost D. Epidemiology of psychological distress In: L’Abate L, editor. Mental illnesses—Understanding, prediction and control. Croatia: InTech (2012). 105–34.

[55] Squires LR Hollett KB Hesson J Harris N. Psychological distress, emotion dysregulation, and coping behaviour: a theoretical perspective of problematic smartphone use. Int J Ment Health Addiction. (2021) 19:1284–99. doi: 10.1007/s11469-020-00224-0

[56] Chen IH Pakpour AH Leung H Potenza MN Su JA Lin CY. Comparing generalized and specific problematic smartphone/internet use: longitudinal relationships between smartphone application-based addiction and social media addiction and psychological distress. J Behav Addict. (2020) 9:410–9. doi: 10.1556/2006.2020.00023, PMID: 32592655 PMC8939406

[57] Volungis AM Kalpidou M Popores C Joyce M. Smartphone addiction and its relationship with indices of social-emotional distress and personality. Int J Ment Health Addiction. (2020) 18:1209–25. doi: 10.1007/s11469-019-00119-9

[58] Stevens C Zhang E Cherkerzian S Chen JA Liu CH. Problematic internet use/computer gaming among US college students: prevalence and correlates with mental health symptoms. Depress Anxiety. (2020) 37:1127–36. doi: 10.1002/da.23094, PMID: 32939888 PMC8635392

[59] Griffiths MD Kuss DJ King DL. Video game addiction: past, present and future. Curr Psyciatry Rev. (2012) 8:308–18. doi: 10.2174/157340012803520414

[60] Jiang L Tieqiao L Yueheng L Hao W Maurage P Billieux J. Prevalence and correlates of problematic online gaming: a systematic review of the evidence published in Chinese. Curr Addict Rep. (2018) 5:359–71. doi: 10.1007/s40429-018-0219-6

[61] Stevens MW Dorstyn D Delfabbro PH King DL. Global prevalence of gaming disorder: a systematic review and meta-analysis. Aust N Z J Psychiatry. (2021) 55:553–68. doi: 10.1177/0004867420962851 33028074

[62] Li Q Wang Y Yang Z Dai W Zheng Y Sun Y. Dysfunctional cognitive control and reward processing in adolescents with internet gaming disorder. Psychophysiology. (2020) 57:e13469. doi: 10.1111/psyp.13469, PMID: 31456249

[63] Shin Y-B Kim H Kim S-J Kim J-J. A neural mechanism of the relationship between impulsivity and emotion dysregulation in patients with internet gaming disorder. Addict Biol. (2020) 26:e12916. doi: 10.1111/adb.12916 32365424

[64] Wang L Tian M Zheng Y Li Q Liu X. Reduced loss aversion and inhibitory control in adolescents with internet gaming disorder. Psychol Addict Behav. (2020) 34:484–96. doi: 10.1037/adb0000549, PMID: 31971427

[65] Yiyu C Yue Yu M Zhanyu Y. Influence of internet game addiction on empathy of college students from the perspective of new game era:the mediating role of alexithymia. Chin J Health Psychol. (2020) 28:1268–72. doi: 10.13342/j.cnki.cjhp.2020.08.033

[66] Loewy RL Corey S Amirfathi F Dabit S Fulford D Pearson R. Childhood trauma and clinical high risk for psychosis. Schizophr Res. (2019) 205:10–4. doi: 10.1016/j.schres.2018.05.003, PMID: 29779964 PMC6939986

[67] Kalmakis KA Chandler GE. Health consequences of adverse childhood experiences: a systematic review. J Am Assoc Nurse Pract. (2015) 27:457–65. doi: 10.1002/2327-6924.12215 25755161

[68] Taylor SE Way BM Seeman TE. Early adversity and adult health outcomes. Dev Psychopathol. (2011) 23:939–54. doi: 10.1017/S0954579411000411 21756443

[69] Felitti VJ Anda RF Nordenberg D Williamson DF MS, PhD Spitz AM MS, MPH Edwards V BA. Relationship of childhood abuse and household dysfunction to many of the leading causes of death in adults. The adverse childhood experiences (ACE) study. Am J Prev Med. (1998) 14:245–58. doi: 10.1016/S0749-3797(98)00017-8, PMID: 9635069

[70] Norman RE Byambaa M de R Butchart A Scott J Vos T. The long-term health consequences of child physical abuse, emotional abuse, and neglect: a systematic review and meta-analysis. PLoS Med. (2012) 9:e1001349. doi: 10.1371/journal.pmed.1001349, PMID: 23209385 PMC3507962

[71] Dong X Zhang R Zhornitsky S le TM Wang W Li CSR. Depression mediates the relationship between childhood trauma and internet addiction in female but not male Chinese adolescents and young adults. J Clin Med. (2021) 10:5015. doi: 10.3390/jcm10215015, PMID: 34768534 PMC8584624

